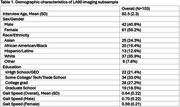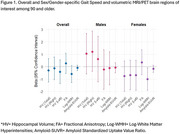# Associations of Gait Speed with Neuroimaging Biomarkers: Findings from the LifeAfter90 (LA90) Study

**DOI:** 10.1002/alz70856_106701

**Published:** 2026-01-07

**Authors:** Rifat B. Alam, Hilary L. Colbeth, Batool M. Rizvi, Alexander Ivan B. Posis, Yi Lor, Kristen M. George, Paola Gilsanz, María M. M. Corrada, Rachel A. Whitmer

**Affiliations:** ^1^ University of California, Davis, Davis, CA, USA; ^2^ Kaiser Permanente Northern California Division of Research, Pleasanton, CA, USA; ^3^ University of California, Irvine, Irvine, CA, USA

## Abstract

**Background:**

Gait speed is a well‐known marker of cognitive function and mortality in older adults. However, its association with neuroimaging biomarkers is understudied. We tested associations between gait speed and biomarkers of neurodegeneration, cerebrovascular injury and amyloid accumulation in the oldest old.

**Method:**

The LifeAfter90 Study (2018‐2022) included a neuroimaging subsample of 103 participants aged 90 and older. Gait speed (meters/second) was measured twice per visit using the 4‐Meter Walk Test and we used the average gait speed score from the visit closest to each participant's neuroimaging scan date. Hippocampal volumes (total, right and left hippocampus) and measures of white matter integrity (log‐white matter hyperintensities (WMH), fractional anisotropy (FA)) were measured with 3T MRI and amyloid standardized uptake value ratio (SUVR) with florbetapir PET. Hippocampal volumes and WMH were adjusted for intracranial volume. Linear regression models tested associations between gait speed and brain imaging markers, adjusting for age, sex/gender, race/ethnicity, and education. Sex‐stratified models were used to explore potential differences in these associations by sex.

**Result:**

Mean age of participants was 92.5(±2.3) years, and 59.2% were women (Table 1). Overall, gait speed was not significantly associated with any brain regions of interest (Figure 1). Although not statistically significant, faster gait speed suggested better white matter integrity (β_logWMH_ = ‐0.59, 95% CI ‐1.28, 0.10; β_FA_ =0.27, 95% CI ‐0.54, 1.09) and lower amyloid‐SUVR (β_amyloid_= ‐0.10, 95% CI ‐0.33, 0.12). In contrast, it was associated with lower hippocampal volumes (β_total_ = ‐0.30, 95% CI ‐1.24, 0.64; β_right_ = ‐0.13, 95% CI ‐1.04, 0.79; β_left_ = ‐0.41, 95% CI ‐1.37, 0.55) (Figure 1). In the stratified analysis, faster gait speed was significantly associated with lower WMH volume in females (β_logWMH_ = ‐1.00, 95% CI ‐1.91, ‐0.10) but not in males (β_logWMH_ = ‐0.12, 95% CI ‐1.46, 1.21).

**Conclusion:**

Although gait speed was not associated with specific neuroimaging biomarkers in the overall cohort, faster gait speed was associated with lower WMH in females. This study underscores the need to further investigate gait speed as an accessible indicator of neuropathological changes, especially in 90+ populations where neuroimaging might be challenging.